# Parallel genomic analysis from paired bone marrow and peripheral blood samples of 200 cytopenic patients

**DOI:** 10.1038/s41375-024-02297-5

**Published:** 2024-05-28

**Authors:** Sandra Huber, Natalie Wossidlo, Torsten Haferlach, Stephan Hutter, Wencke Walter, Christian Pohlkamp, Isolde Summerer, Henning Ruge, Constance Baer, Gregor Hoermann, Manja Meggendorfer, Wolfgang Kern, Claudia Haferlach

**Affiliations:** MLL Munich Leukemia Laboratory, Max-Lebsche-Platz 31, Munich, 81377 Germany

**Keywords:** Cancer genomics, Cytogenetics

## To the Editor:

Bone marrow (BM) analysis is required for morphological and genetic assessment in patients evaluated for myelodysplastic neoplasms (MDS) and thus currently represents the gold standard in patients with unexplainable cytopenia [1, 2]. Since frequent BM sampling is an invasive procedure with a low but notable rate of complications, often perceived as painful and inconvenient [3], transitioning to analyzing peripheral blood (PB) would be a significant advancement, particularly for the elderly. Previously, a high concordance regarding mutational information between BM and PB has been shown (reviewed in [4]). Unlike mutational concordance between BM and PB, little is known whether cytogenetic information covering the entire genome can reliably be retrieved from PB [5–7]. Chromosome banding analysis (CBA) of BM metaphases represents the gold standard for cytogenetic diagnostics while karyotyping from PB is frequently limited by cytopenia, low blast count, or lack of in vitro proliferation [6]. The aim of this study was to analyze somatic mutations and copy number variations (CNV) in concurrent PB and BM samples in cytopenic patients using comprehensive NGS-based analyses to omit the need for frequent BM sampling.

We analyzed paired BM and PB samples (sampled within 14 days) of 200 cytopenic patients (female/male: 41%/59%; median age: 72 [28–93] years; Supplementary Table S1). The cohort comprised 75 patients with confirmed MDS according to WHO 2022 classification and 125 with suspected MDS (52 finally diagnosed as CCUS, 43 as ICUS, 30 without final diagnosis). All patients gave their written informed consent, the study was approved by the institutional review board and conducted according to the Declaration of Helsinki. All samples were subjected to targeted panel sequencing analyzing mutations in 40 myeloid genes. For analyzing CNV from PB, the xGen™ Human Copy Number Variant Backbone Hybridization Panel was added. Details on sequencing and statistics are described in the Supplement.

**Fig. 1:**
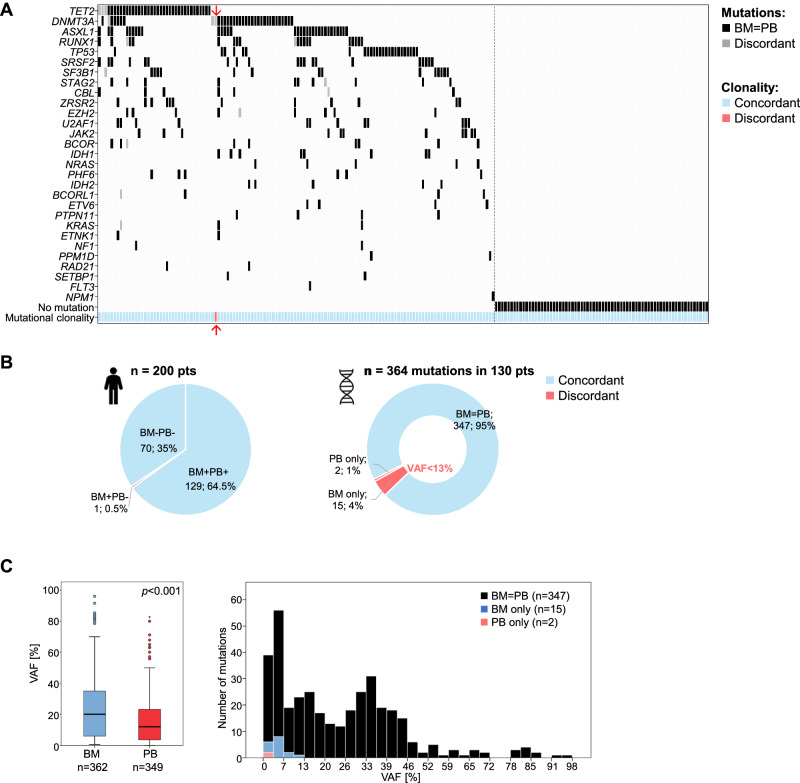
Mutational analysis comparing BM and PB. **A** Illustration of all 200 patients, each column represents one patient. Mutations were detected in BM and PB (black: BM = PB) or either BM only or PB only (grey: discordant). Arrow indicates patient whose sole *DNMT3A* mutation was detected in BM only. **B** Comparison of mutational clonality detection in BM and PB of the 200 patients (left) and comparison of individual mutation detection in 130 patients with mutations (right); light blue: concordant; red: discordant. BM bone marrow; PB peripheral blood; pts: patients; “+”: positive; “–”: negative; VAF variant allele frequency. **C** Analysis of variant allele frequency of detected mutations depicted by a boxplot (left) showing the distribution and median of VAF of mutations detected in BM or PB and by a histogram (right) showing the VAF of mutations and their detection in BM and PB (black), BM only (blue) or PB only (red). BM VAF is shown in the histogram except for PB only cases (n = 2); n: number of mutations; VAF variant allele frequency.

In 70 patients (35%) mutations were detected neither in BM nor PB (Fig. 1; Supplementary Fig. S1). For the remaining 130 patients at least one mutation was found (median mutations/patient: 2; range: 1–10; Supplementary Results; Supplementary Fig. S2). Mutations were detected in both BM and PB in 129/130 patients. The same number of mutations were found in 118 patients in BM and PB, while more mutations in BM were detected in 9 and more mutations in PB in 2 patients (Supplementary Fig. S1). Overall, we detected 364 mutations in 29 of the analyzed genes with *TET2* (15%), *DNMT3A* (10%), and *ASXL1* (9%) most frequently mutated (Fig. 1; Supplementary Fig. S2). The median VAF was lower in PB compared to BM across all mutations (12% vs. 20%; *p* < 0.001; median BM-PB VAF difference: 4%; Fig. 1C; a detailed evaluation is provided in the Supplementary Results/Supplementary Figs. S3/S4/S5). Of all detected mutations, 347 (95%) were found in both BM and PB, while 15 (4%) and two (1%, both *DNMT3A*) mutations were only detected in BM or PB, respectively (Fig. 1; Supplementary Table S2). The high concordance of 95% on individual mutation level is in line with previous studies [4]. Of note, all 17 discordant mutations were found in small clones with the BM-only mutations harboring VAF below 13% and the PB-only mutations below 5% (Fig. 1C; Supplementary Table S2), indicating that the discrepancies in mutation detection was caused by low-level clonal events. Even though 15 individual mutations from 10 patients were missed in PB analysis, it was still possible to identify clonality in 9 of these patients. Thus, the finding of at least one somatic mutation, an important diagnostic marker of clonality, was consistent between PB and BM in all but one patient (199/200; 99.5%). Only one CCUS patient showed no mutations in PB while harboring a *DNMT3A* mutation as the sole mutation in BM (VAF: 9%, Fig. 1). Lack of mutations was correctly identified from PB in 70/71 patients resulting in a negative predictive value (NPV) of 98.6% and a positive predictive value (PPV) of 100% (129/129) for the detection of mutational clonality, meaning the detection of any somatic mutations, in PB compared to BM, which corresponds to a clinical sensitivity of 99.2% and specificity of 100%.

**Fig. 2:**
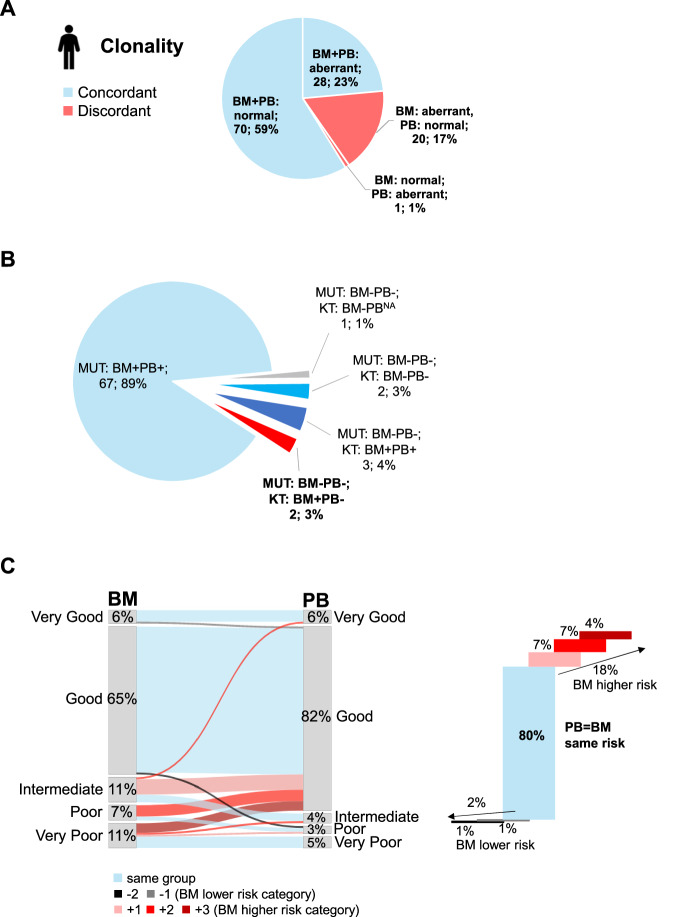
Cytogenetic analysis comparing BM and PB and clonality detection in confirmed MDS cases. **A** Comparison of cytogenetic clonality detection in BM and PB of 119 patients; light blue: concordant; red: discordant. **B** Evaluation of detection of clonality comparing BM and PB of patients with confirmed MDS (n = 75). MUT: mutation; “+”: positive/aberrant; “–”: negative/normal; KT: karyotype; NA: not available; blue colors: concordant; red: discordant. **C** IPSS-R cytogenetic risk group assessment of 119 patients comparing PB with BM (left panel). Differences in risk groups between PB and BM are indicated by colors and quantitatively evaluated in the right panel; Patients that differed by three IPSS-R cytogenetic categories all harbored a complex BM karyotype. light blue: same risk group; black/grey (-): lower risk group in BM; pink/ red/ dark red (+): higher risk group in BM.

Next, we focused on the detection of cytogenetic abnormalities. Therefore, we compared BM CBA with PB NGS CNV detection in a subset of 119 patients. BM CBA revealed a normal karyotype in 71 and an aberrant karyotype in 48 patients (Supplementary Figs. S6/S7). The proportion of aberrant karyotypes was higher in patients with confirmed MDS (71%) than in those without (20%). In 88/119 patients (74%) concordant results were found with both BM and PB showing either normal (n = 70) or aberrant karyotypes (n = 18), while the remaining 31 patients (26%) showed discordant results (Supplementary Figs. S6/S7; Tables S3/S4). Hence, regarding cytogenetic clonality, meaning the detection of any cytogenetic abnormalities, concordant results in BM and PB were found in 82% (98/119) of patients, while discordant results were seen in 21 patients (18%) (Fig. 2A). This resulted in a NPV of 78% (71/91) and a PPV of 98% (48/49) for the detection of cytogenetic clonality in PB NGS compared to BM karyotype. Details are described in the Supplements (Supplementary Results; Supplementary Figs. S8/9; Supplementary Table S4). Overall, we found that discrepancies in CNV detection can be accounted to more sensitive detection of abnormalities in CBA due to a proliferative advantage of the abnormal clone and due to smaller clone sizes in PB compared to BM supported by mutational analysis. In cases with large differences in clone size between BM and PB CD34-enriched PB samples might help to overcome these limitations [6–8].

Then, mutational and cytogenetic results were combined for evaluating the overall detection of clonality in all 200 cytopenic patients. Of note, in 80% (16/20) of cytogenetically false-negative cases, mutational clonality was reliably detected in PB. The overall incidence of clonality was independent of the type of cytopenia (Supplementary Figs. S10/11) but was associated with the degree of anemia (HB < 9 g/dL; 87% vs. 67%; *p* = 0.02), the number of cytopenias (logistic regression; *p* = 0.025), and BM blasts (64% vs. 91% for <5% vs. ≥5% blasts; *p* < 0.001). We further evaluated the detection of clonality in patients with confirmed MDS (n = 75) separately. Mutational clonality was detected in PB and BM in 89% (n = 67), whereas 8 (11%) MDS patients did not harbor any somatic mutation neither in BM nor PB (100% concordance; Fig. 2B). Of these 8 patients, 5 had aberrant BM karyotypes as the sole indicator of clonality without any somatic mutation (6.7%). Two of these patients with aberrant BM karyotype showed a normal pattern in PB NGS (Supplementary Table S3). Thus, from 75 MDS patients only in two (2.7%) clonality would have been missed by analyzing PB compared to BM.

In the last step, we analyzed the IPSS-R cytogenetic risk group retrieved from PB NGS compared to BM karyotype as this parameter is considered in several risk models. A slight skewing towards lower risk groups was detected in PB compared to BM (Fig. 2C). However, 80% of patients (n = 95) fell into the same cytogenetic risk group, 18% (n = 22; all with aberrant BM karyotype) were assigned to a better group according to PB NGS compared to BM and 2% (n = 2) were assigned to a worse risk group in PB, respectively (Fig. 2C; Supplementary Tables S5/S6). Notably, in 10/24 discordant cases, the IPSS-R cytogenetic risk group differed only by one category. In contrast to the pure cytogenetic risk group, the concordance of the full IPSS-R or IPPS-M risk category was substantially lower when also results from BM blast count were omitted (Supplementary Fig. S13). Likewise, an increase of blasts in MDS was more often seen in BM compared to PB (Supplementary Table S1) also arguing for a relevance of BM assessment in MDS risk stratification. In particular, down-staging in PB could potentially impact treatment decisions.

Our current detection limit for CNV by NGS is a clone size of 30% as validated by comparison to FISH and the method is not suitable for detecting balanced structural variants. However, individual CNV discrepancies impacted the finding of genetic clonality in PB only to a low extent. Other limitations of the study include the retrospective design and the lack of follow-up information. Thus, prospective studies are required for monitoring cytopenic patients in real-time to further validate the suitability of PB for clonality detection as well as clonal expansion over time.

In summary, our data indicate a high informative value of one single NGS-based approach from PB in patients with unexplained cytopenia. This approach might be applied as the first screening method omitting the need for BM sampling in cases with low pre-test probability for MDS (Supplementary Fig. S14). When clonality is detected in PB, current guidelines recommend a BM examination for diagnosing a myeloid neoplasm [9] and assessing prognostic scores [10]. BM examination at diagnosis might be replaced in the future by PB monitoring in certain clinical settings (e.g., small/stable clone, low-risk mutation like sole *DNMT3A* [11], no therapeutic indication) [12–14]. In addition, in the setting of a BM-confirmed CCUS/MDS diagnosis, NGS analysis from PB may be considered for disease monitoring in cases without clinical progression [4].

### Supplementary information

Supplement (clean)

## Data Availability

The datasets generated during and/or analyzed during the current study are available from the corresponding author on reasonable request.
