# Supplementary material for: Parallel genomic analysis from paired bone marrow and peripheral blood samples of 200 cytopenic patients
Source: Leukemia. 2024 May 28;38(7):1626–9. doi: 10.1038/s41375-024-02297-5 (PMC11216983; doi:10.1038/s41375-024-02297-5)
Supplement: Supplementary file 1 — Supplement (clean) [file 41375_2024_2297_MOESM1_ESM.docx]

**Supplementary Material**

***Patient cohort and samples***

Samples were sent to the MLL Munich Leukemia Laboratory between 02/2017 and 03/2023. If applicable, diagnoses (from PB and BM) were made based on cytomorphology, cytogenetics (including chromosome banding analysis/CBA and FISH), immunophenotyping and molecular genetics as previously published [1-3]. The cohort included 103 patients with BM and PB sampled on the same day and 97 patients with both material sampled within 2 weeks (median 7 [1-14] days).

**Supplementary Methods**

***Targeted panel NGS***

Targeted panel sequencing (coverage: 1500x) was performed as previously described [4]. In brief, for DNA isolation, the MagNA Pure 96 system (Roche Diagnostics, Mannheim, Germany) was used. Sequencing was performed on NextSeq or NovaSeq platforms after TruSeq Custom Amplicon Low Input library preparation or Nextera DNA Flex library preparation (all Illumina, San Diego, CA) and target enrichment with a custom panel by IDT (Integrated DNA Technologies, Iowa, USA) following manufacturer’s instructions. Gene variants were called with Pisces (BaseSpace, Illumina) using a sensitivity level of 3% variant allele frequency (VAF). Variants with VAF below the sensitivity level in the corresponding sample were manually reviewed using the integrative genomics viewer (version 2.4.8 [5]) and considered as confirmed at a VAF of 1%. Individual variants that were called in only one of the corresponding samples were reviewed likewise. For variant interpretation following a four-tier classification [6] several databases (COSMIC, IRAC and ClinVAR) and in silico predictions were used. Only pathogenic or likely pathogenic mutations were considered, excluding putative germline variants and single nucleotide polymorphisms from the data analysis. In this study, we evaluated mutations in 40 genes associated with myeloid neoplasms for all patients (*ASXL1, ASXL2, BCOR, BCORL1, BRAF, CALR, CBL, CSF3R, CSNK1A1, DNMT3A, ETNK1, ETV6, EZH2, FLT3, GATA1, GATA2, IDH1, IDH2, JAK2, KIT, KRAS, MPL, NF1, NOTCH1, NPM1, NRAS, PHF6, PIGA, PTPN11, RAD21, RUNX1, SETBP1, SF3B1, SRSF2, STAG2, TET2, TP53, U2AF1, WT1, ZRSR2*).

***CNV analysis using NGS***

For analyzing CNV from PB (available in n=119), the xGen™ Human Copy Number Variant Backbone Hybridization Panel was added to the custom panel (spike-in ratio 1:8, coverage: ~50x). CNV were called using CNVkit (version 0.9.6 [7]). The analytical sensitivity of the method (determined for del(17p)) is a clone size of 30%.

***Risk prediction***

The IPSS-R and IPSS-M were calculated as previously described [8, 9]. *KMT2A*-PTD was analyzed with Mentype AMLplex screening assay (BIOTYPE GmbH, Dresden, Germany) following manufacturer's instructions. Genes required for IPSS-M calculation that were not analyzed in this study were set as negative in both BM and PB. For calculating the IPSS-M in PB samples, BM blasts were assumed to be 0%.

***Statistics***

For statistical analyses SPSS version 19.0 (IBM Corporation, Armonk, NY) and R software version 4.3.0 (R Foundation for Statistical Computing, Vienna, Austria) were used. Dichotomous variables were compared using Fishers exact or chi-square test. For comparison of median values, the two-sided Mann-Whitney U test was applied. All reported *p*-values are two-sided and were considered significant at *p*≤0.05.

**Supplementary Results**

***Mutational analysis – clonality* depending on diagnosis**

Mutational clonality was observed more frequently in patients with confirmed MDS compared to those without MDS diagnosis (Suppl. Figure S2).

***Mutation analysis – variant allele frequency (VAF) depending on diagnosis***

A higher median VAF in BM compared to PB was observed in both confirmed and non-confirmed MDS cases (Suppl. Figure S4). In confirmed MDS cases the median VAF in both BM and PB was significantly higher compared to non-confirmed MDS cases (27% and 15% compared to 13% and 9%, respectively; *p*=0.001 and *p*=0.044). Confirmed MDS patients also harbored significantly more mutations than non-confirmed MDS patients (median BM mutations: 3 [0-10] vs. 1 [0-10]; *p*<0.001; Suppl. Figure S4E).

***Mutation analysis – sampling time interval***

The proportion of concordant mutations, meaning the reliable detection in both BM and PB, was higher in patients when BM and PB were sampled on the same day (Suppl. Figure S5A+B). A higher median VAF in BM compared to PB was observed in all cases independent of sampling time interval (same day sampling: 16% vs. 10%, *p*=0.001; sampling within 1-14 days: 27% vs. 13%, *p*<0.001; Suppl. Figure S5C+D).

***Cytogenetic analysis – aberration level***

In total, 130 aberrations were detected of which 64 (49%) were concordant between BM CBA and PB NGS (Suppl. Figure S8). From the 66 discordant aberrations 7 were balanced SV that can technically not be detected using the CNV backbone panel, 4 were detected only in PB with NGS indicating that those were missed by CBA as these clones did not grow *in vitro* and additional information is provided here by PB NGS (Suppl. Table S4). 55 CNV were exclusively found in BM CBA (of 26 patients). Notably, in the CNV analysis we have two influencing factors (i) the different material and (ii) also the different techniques with the impact of proliferation in CBA. Thus, for the cases in which CNV were only found in BM CBA but not in PB NGS we additionally performed NGS and FISH on BM which are both independent of proliferation. This was done to analyze whether in these cases the discordant results were due to the different material or due to the different techniques assessing different cell compartments (all cells vs. proliferating cells only). Of the 55 CNV detected in BM CBA but not in PB NGS, 53% (n=29) were detected in BM NGS indicating differences between BM and PB suggesting that the proportion of aberrant cells in PB was lower than in BM and thus slipped below the detection limit of CNV NGS (Suppl. Figure S8). This is supported by data from the mutational analysis showing a very large difference of mutational VAF between BM and PB especially in these cases in which CNV were only detected in BM but not in PB using NGS (median BM-PB VAF difference: 13%; Suppl. Figure S8). In 47% (26/55) CNV were also not detected by NGS in the BM as their clone sizes measured by FISH were below the detection limit and only seen in CBA due to high *in vitro* proliferation (Suppl. Figure S8A). In summary, CNV detection by NGS from PB is reasonable and reliable if the proportion of aberrant cells is larger than ~30% and comparable in BM and PB (Suppl. Figure S8B). Focusing on specific (MDS-associated) chromosomal abnormalities, del(5q) was detected in PB in 100% (7/7) if the close size measured by BM FISH was larger than 30% (7/10) (Suppl. Figure S9). Trisomy 8 was only detected in 3/11 cases in PB. However, trisomy 8 was often (6/8 cases with available BM FISH) found in small clones (<30% BM FISH) and if not detected in PB, it was also not detected in BM by NGS. Monosomy 7 (n=7) was found in PB by NGS in 3 cases, while in the remaining cases, although not seen in PB, the aberration was nevertheless detected by BM NGS indicating that in these cases the clone size in PB was much smaller than in BM and below the detection limit in PB.

***Combined evaluation of mutations and copy number variations for clonality detection in suspected MDS cases***

For 125 patients without a confirmed MDS diagnosis, 62 (50%) showed mutational clonality in PB and BM, while in one patient (0.8%) PB analysis did not capture the mutational clonality of the BM (Suppl. Figure S12). Of the mutation-negative cases (BM and PB; n=62) CNV analysis from PB was available in 35 patients. Of these, 31 had a normal BM karyotype and showed a normal pattern in PB NGS. Of the four patients with an aberrant BM karyotype PB NGS detected cytogenetic abnormalities in two patients while in the other two patients no abnormalities were detected in PB (potentially congenital CNV or small clone; Suppl. Table S3).

**Supplementary Tables and Figures**

**Table S1. Cohort overview**

| Characteristics | All patients (n = 200) | |
| --- | --- | --- |
| **Age** (years; median [range]) | 72 [28-88] | |
| **Sex** (female/male) | 82 (41%) / 118 (59%) | |
| **BM + PB sampling** (same day/<15 days) | 103 (51.5%) / 97 (48.5%) | |
| **PB with CNV backbone** - availability | 119 (60%) | |
| **Cytopenias** (3; 2; 1; unclear) | 56 (28%); 70 (35%); 52 (26%); 22 (11%) | |
| **Anemia** (♂ HB <13 g/dL, ♀ HB <12 g/dL) | 142/173 (82%) | |
| **Thrombocytopenia** (PLT <150x10^9^/L) | 122/174 (70%) | |
| **Neutropenia** (ANC <1.8x10^9^/L) | 94/175 (54%) | |
| **HB** (g/dl; median; IQR): n=154 | 9.9 (8.5 – 11.7) | |
| **Thrombocytes / µl** (median; IQR): n=155 | 110,000 (57,500 – 176,500) | |
| **Neutrophiles / µl** (median; IQR): n=131 | 1,620 (938 – 2,497) | |
| **Confirmed MDS diagnosis (n)** | 75 (38%) | |
| BM blasts ≥5% | 41/75 (55%) | |
| PB blasts ≥1% | 27/75 (36%) | |
| Morphologic dysplasia in MDS patients | positive / cases with available data | |
| Dysplasia in granulopoiesis | 55/66 (83%) | |
| Dysplasia in erythropoiesis | 52/64 (81%) | |
| Dysplasia in megakaryopoiesis | 39/59 (66%) | |
| Dysplasia in 3 lineages | 30/66 (45%) | |
| Dysplasia in 2 lineages | 17/66 (26%) | |
| Dysplasia in 1 lineage | 12/66 (18%) | |
| Dysplasia in ≥ 1 lineage * | 7/66 (11%) | |
| **MDS not confirmed (n)** | 125 (62%) | |
| BM cytomorphology (n) | available  99 | not available  26 |
| Clonality (n) | 52 (CCUS) | 16 |
| No clonality (n) | 43 (ICUS) | 5 |
| No MUT clonality (cytogenetics NA) (n) | 4 | 5 |

BM: bone marrow; PB: peripheral blood; HB: hemoglobin; PLT: platelets; ANC: absolute neutrophile count; MUT: mutational; NA: not available; * no data of all lineages available

**Table S2. Discordant mutations**

|  | **Gene** | **Position** | **BM VAF [%]** | **PB VAF [%]** | **Patients (pts)** | **#MUT/pts** |
| --- | --- | --- | --- | --- | --- | --- |
| **BM only** | ***TET2*** | c.2911G>T | 1.4 | Not detected* | 1 | BM>PB |
|  | ***CBL*** | c.1199_1200delinsAT | 2.4 | Not detected* | 2 | BM>PB |
|  | ***BCORL1*** | c.3529_3530del | 2.9 | Not detected* | 3 | BM>PB |
|  | ***KRAS*** | c.179G>A | 6.1 | Not detected* |  |  |
|  | ***SF3B1*** | c.2098A>G | 3.5 | Not detected* | 4 | BM>PB |
|  | ***TET2*** | c.2488dup | 3.7 | Not detected* |  |  |
|  | ***EZH2*** | c.1876G>A | 3.9 | Not detected* | 5 | BM>PB |
|  | ***BCOR*** | c.3649C>T | 3.8 | Not detected* | 6 | BM>PB |
|  | ***RUNX1*** | c.415C>G | 3.6 | Not detected* |  |  |
|  | ***RUNX1*** | c.341_360del | 3.8 | Not detected* |  |  |
|  | ***STAG2*** | c.3361_3362ins | 4.5 | Not detected* | 7 | BM>PB |
|  | ***RUNX1*** | c.339_340insCC | 6.2 | Not detected* | 8 | BM>PB |
|  | ***RUNX1*** | c.363del | 6.7 | Not detected* |  |  |
|  | ***TET2*** | c.1630C>T | 12.1 | Not detected* | 9 | BM>PB |
|  | ***DNMT3A*** | c.2033del | 9.0 | Not detected* | 10 | BM+/PB- |
| **PB only** | ***DNMT3A*** | c.875T>C | Not detected* | 3.2 | 11 | BM<PB |
|  | ***DNMT3A*** | c.1585G>A | Not detected* | 3.8 | 12 | BM<PB |

VAF: variant allelic frequency; MUT: mutation; *below 1% VAF; # number; ”+”: positive; ”-”: negative; (Sequencing was performed with a median coverage of 1000x and a minimum coverage of 400x to ensure the detection of small clones. The genomic regions of each non-detected variant met these criteria, irrespective of the material. Manual review of the respective variants revealed the presence of the mutations in some reads, but in less than 1% and thus below the limit of detection of the assay applied. Hence, non-detected variants were most likely due to lower VAF in the respective material.)

**Table S3. Patients with BM clonality not detected in PB**

| Diagnosis | Age (gender) | BM blasts (%) | PB blasts (%) | BM MUT | PB MUT | BM KT (CBA) | PB CNV (NGS) |
| --- | --- | --- | --- | --- | --- | --- | --- |
| Sus. MDS | 44 yrs (♀) | 1 | 0 | *DNMT3A*  9% | negative | 46,XX[20] | n.a.d. |
| Sus. MDS | 68 yrs (♂) | 2 | 0 | negative | negative | 45,XY,der(14;21)(q10;q10)[20]  (potentially congenital) | n.a.d. |
| Sus. MDS | 75 yrs (♀) | 2 | 0 | negative | negative | 47,XX,+8[4]/46,XX[2]  (+8 in FISH: 2.5%) | n.a.d. |
| MDS | 85 yrs (♂) | 16 | 2 | negative | negative | 46,XY,t(3;21)(q26;q22)[15]/  47,XY,t(3;21)(q26;q22),+9[2]/  46,XY[8] | n.a.d. |
| MDS | 41 yrs (♂) | 8 | 0 | negative | negative | 46,XY,t(4;12)(q22;q21)[17]/  46,XY[3] | n.a.d. |

Sus.: Suspected/ not confirmed; MUT: mutation; KT: karyotype; CBA: chromosome banding analysis; NGS: next generation sequencing; n.a.d.: no abnormality detected

**Table S4. CNV detected in PB only**

| Diagnosis | Age (gender) | BM KT (CBA) | PB CNV (NGS) |
| --- | --- | --- | --- |
| Sus. MDS | 83 yrs (♂) | 46,XY,del(20)(q11q13)[7]  47,XY,+8,+del(20)(q11q13),del(20)(q11q13)[11]  46,XY[2] | **del(12q)*,**  del(20q) |
| Sus. MDS | 82 yrs (♂) | 46,XY[20] | **del(7q),**  **+9p**  (+9 in FISH: 30%) |
| MDS | 73 yrs (♀) | 47,XX,+8[2]  46,XX[20] | **del(11q)*** |

Sus.: Suspected/ not confirmed; MUT: mutation; KT: karyotype; CBA: chromosome banding analysis; NGS: next generation sequencing; * no FISH probe available

**Table S5. Patients with discordant IPSS-R cytogenetic risk groups**

| Diagnosis | BM KT (CBA) | PB CNV (NGS) | Risk group BM | Risk group PB | Risk group BM-PB | MUT |
| --- | --- | --- | --- | --- | --- | --- |
| Sus. MDS | Complex 1 (see Table S6) | n.a.d. | 4 | 1 | 3 | BM+PB+ |
| MDS | Complex 2 (see Table S6) | n.a.d. | 4 | 1 | 3 | BM+PB+ |
| MDS | Complex 3 (see Table S6) | n.a.d. | 4 | 1 | 3 | BM+PB+ |
| MDS | Complex 4 (see Table S6) | del(5q) | 4 | 1 | 3 | BM+PB+ |
| Sus. MDS | Complex 5 (see Table S6) | del(5q),  del(6p) | 4 | 1 | 3 | BM+PB+ |
| Sus. MDS | 45,XX,-7[15]  45,XX,-7.ish 3~10dmin(3'MECOM+) [5] | n.a.d. | 3 | 1 | 2 | BM+PB+ |
| MDS | 46,XX,inv(3)(q21q26)[15] | n.a.d. | 3 | 1 | 2 | BM+PB+ |
| MDS | 45,XX,-7[11]  46,XX[9] | n.a.d. | 3 | 1 | 2 | BM+PB+ |
| MDS | 46,XY,del(3)(q13q26)[18]  46,XY[2] | n.a.d. | 3 | 1 | 2 | BM+PB+ |
| MDS | 45,XX,-7[3]  46,XX[9] | n.a.d. | 3 | 1 | 2 | BM+PB+ |
| MDS | Complex 6 (see Table S6) | n.a.d. | 3 | 1 | 2 | BM+PB+ |
| MDS | 47,XX,+8[2]  46,XX[20] | del(11q) | 2 | 0 | 2 | BM+PB+ |
| Sus. MDS | Complex 7 (see Table S6) | +1q | 4 | 2 | 2 | BM+PB+ |
| MDS | 46,XY,t(4;12)(q22;q21)[17]  46,XY[3] | n.a.d. | 2 | 1 | 1 | **BM-PB-** |
| Sus. MDS | 47,XY,+8[8]  46,XY[12] | n.a.d. | 2 | 1 | 1 | BM+PB+ |
| MDS | 46,XX,+1,der(1;7)(q10;p10)[1]  46,XX[19] | n.a.d. | 2 | 1 | 1 | BM+PB+ |
| Sus. MDS | 47,XX,+8[4]  46,XX[2] | n.a.d. | 2 | 1 | 1 | **BM-PB-** |
| MDS | 46,XY,t(3;21)(q26;q22)[15]  47,XY,t(3;21)(q26;q22),+9[2]  46,XY[8] | n.a.d. | 2 | 1 | 1 | **BM-PB-** |
| Sus. MDS | 45,XY,der(14;21)(q10;q10)c or 45,XY,der(14;21)(q10;q10)[20] | n.a.d. | 2 | 1 | 1 | **BM-PB-** |
| MDS | 46,XY,+1,der(1;7)(q10;p10)[10]  46,XY[12] | n.a.d. | 2 | 1 | 1 | BM+PB+ |
| MDS | 47,XX,+8[6]  46,XX[15] | n.a.d. | 2 | 1 | 1 | BM+PB+ |
| MDS | Complex 8 (see Table S6) | n.a.d. | 4 | 3 | 1 | BM+PB+ |
| MDS | 45,X,-Y[14]  46,XY[6] | n.a.d. | 0 | 1 | -1 | BM+PB+ |
| Sus. MDS | 46,XY[20] | del(7)(q33q36), +9(p21p24) | 1 | 3 | -2 | BM+PB+ |

Sus.: Suspected/ not confirmed; MUT: mutation (”+”: positive; ”-”: negative); KT: karyotype; CBA: chromosome banding analysis; NGS: next generation sequencing; n.a.d.: no abnormality detected

**Table S6. Complex BM karyotypes of Table S5**

| Sample | BM KT (CBA) |
| --- | --- |
| Complex 1 | 43,XY,del(5)(q14q34),dic(6;17)(p21;p13),der(7;18)(p10;p10),+11,der(14;20)(q10;p10),dic(19;21)(p13;p11) [13]/  46,XY [13] |
| Complex 2 | 47,XY,del(5)(q21q34),+8,+der(11)(::11p11->11q13::11q22->11q24::19?q13->19?q13::),der(11)(::19?q13->19?q13::11q23->11q23::11p11->11q11::11q22->11q23::),-18,der(19)t(11;19)(p13;?q13) [15]/  46,XY [5] |
| Complex 3 | 42,XY,-3,+5,der(5;9)(p10;p10),der(5;21)(p10;q10),-7,der(7;15)(7pter->7q36::15p11->15q26::7q31->7qter) [5]/  43,XY,-3,+5,+der(5;21)(p10;q10),der(5;9)(p10;p10),der(5;21)(p10;q10),-7,der(7;15)(7pter->7q36::15p11->15q26::7q31->7qter) [14]/  46,XY [1] |
| Complex 4 | 46,XX,del(1)(p31p34),del(5)(q14q34),+8,dic(17;18)(p12;p11) [22]/46,XY [1] |
| Complex 5 | 43,XY,der(1;3)(q10;q10),der(5)t(5;7)(q14;q31),dic(6;14)(p21;p11),der(7)t(1;7)(p34;q21),dic(14;22)(p11;p11) [11]/  46,XY [4] |
| Complex 6 | 47,XX,dic(5;8)(q12;p21),+dic(5;8)(q12;p21)x2,der(13)t(13;17)(?q31;q11),der(17)t(13;17)(q33;q11) [15]/  46,XY [5] |
| Complex 7 | 46,XY,+1,der(1;22)(q10;q10) [7]/  46,XY,+1,der(1;22)(q10;q10),del(20)(q11q13) [9]/  48,XY,+1,der(1;22)(q10;q10),+8,+9,del(20)(q11q13) [5] |
| Complex 8 | 44,XX,der(4)t(4;12)(q13;?p13),r(5)(p13q12),r(7)(p11q11),der(11)t(7;11)(?p11;?p14),der(12)del(12)(p13p11)t(12;17)(q11;q11),der(16;17)(q10;p10)del(17)(p13p13),-18,der(19)dup(19)(p13p13)dup(19)(q13q13) [8]/  45,XX,der(4)t(4;12)(q13;?p13),r(5)(p13q12),der(7)t(4;7)(q28;q22),der(12)del(12)(p13p11)t(12;17)(q11;q11),der(16;17)(q10;p10)del(17)(p13p13),del(16)(q12q24),der(18)t(18;19)(q12;p11),del(19)(p13p11) [5]/  44,XX,der(4)t(4;12)(q13;?p13),r(5)(p13q12),r(7)(p11q11),der(11;18)(q10;p10),der(12)del(12)(p13p11)t(12;17)(q11;q11),der(15)t(7;15)(p11;p11),der(16;17)(q10;p10)del(17)(p13p13) [3] |

**Supplementary Figures**

**
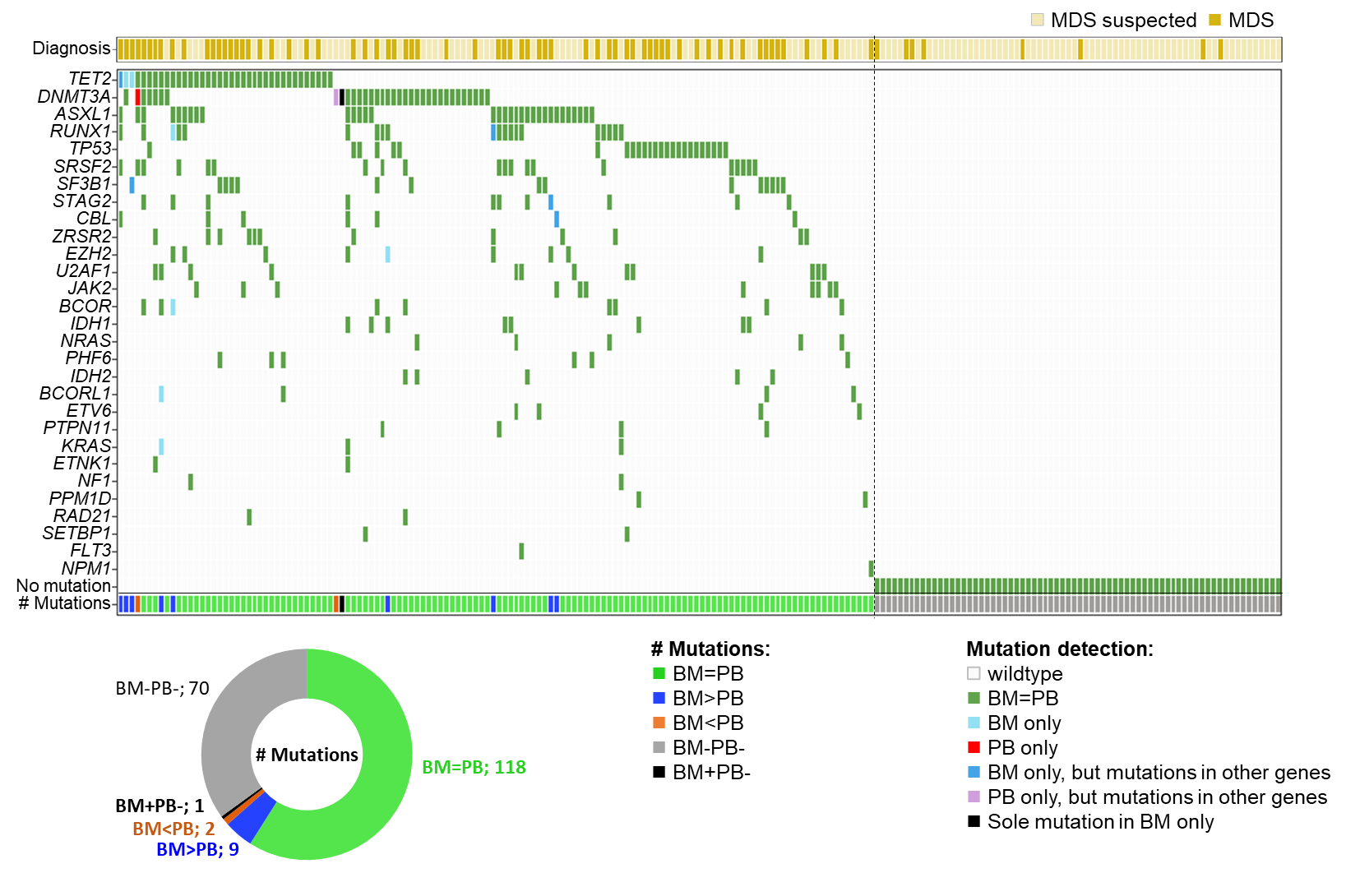
**

**Supplementary Figure S1: Detailed mutational analysis comparing BM and PB.** Illustration of all 200 patients, each column represents one patient. Mutations were detected in BM and PB (green: BM=PB) or either BM only (blue colors) or PB only (red/purple). Detected numbers of (#) mutations were also compared between BM and PB (donut chart).

**
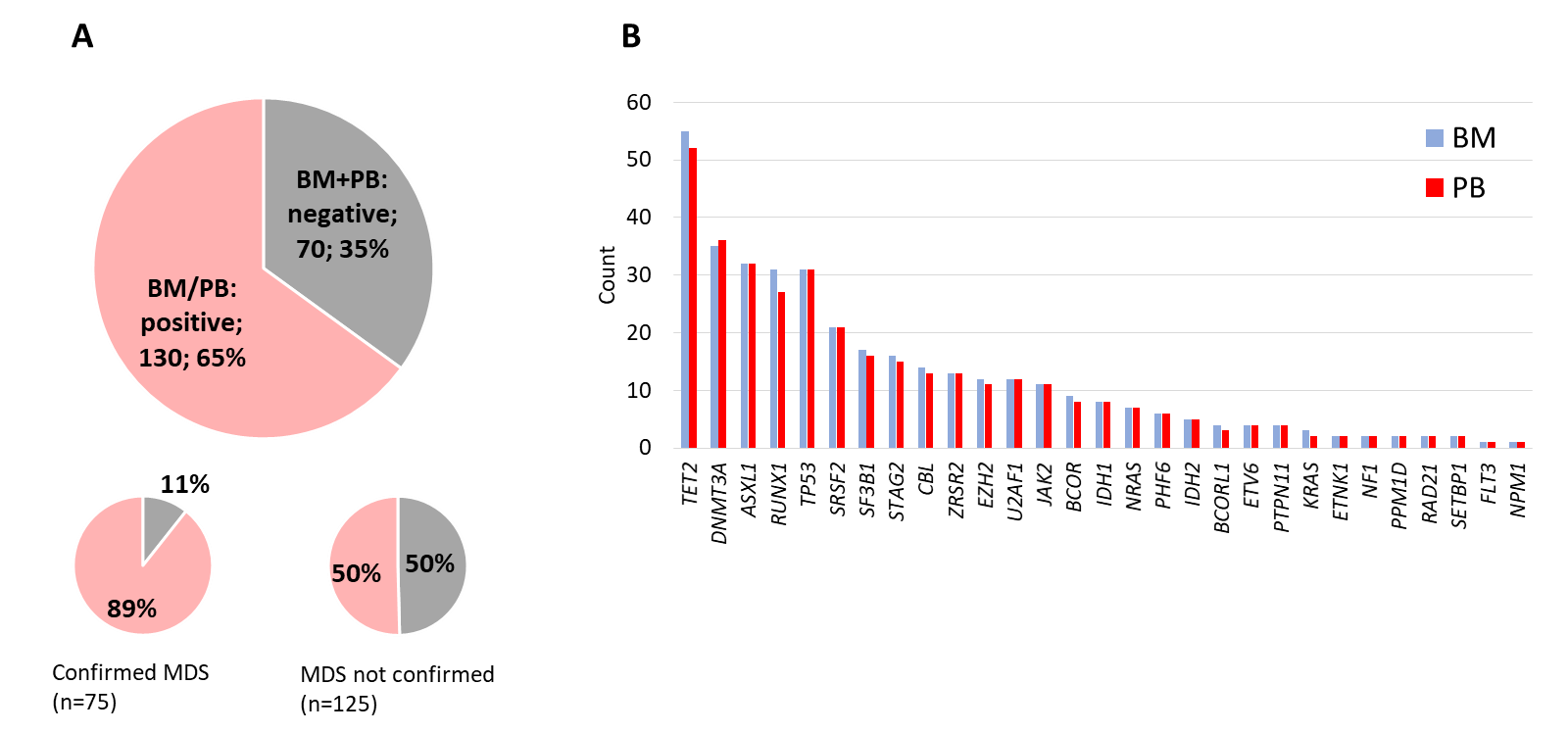
**

**Supplementary Figure S2: Mutational analysis in BM and PB in 200 patients. (A)** Evaluation of patients with mutations detected in BM or PB (pink) and without mutations in BM and PB (grey) for all patients (upper chart) and grouped into patients with and without confirmed MDS diagnosis separately (lower charts). **(B)** Number of mutations detected in BM (blue) and PB (red) in the respective gene.

**
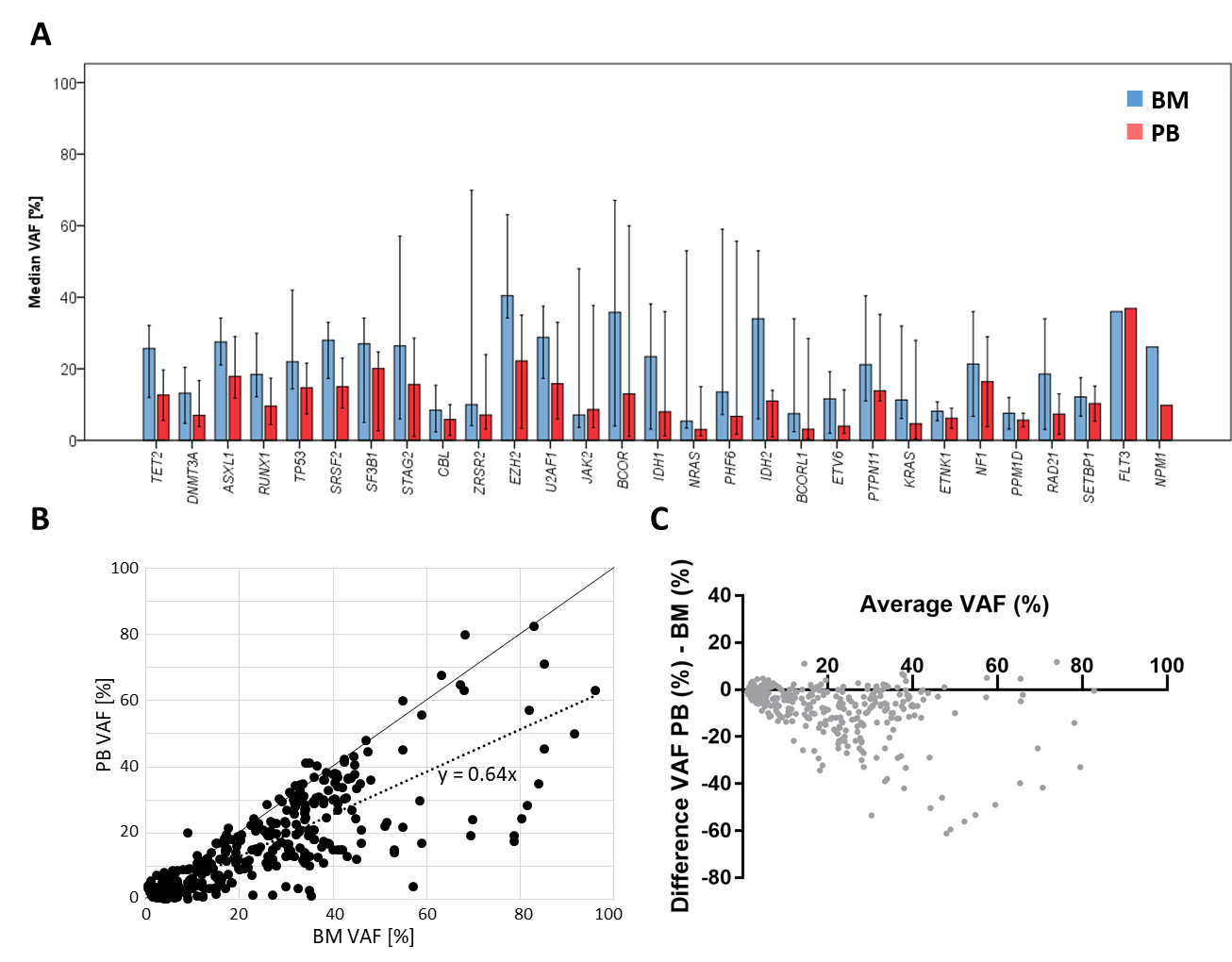
**

**Supplementary Figure S3: Detailed analysis of variant allele frequency of detected mutations. (A)** Median VAF of mutations in the respective gene detected in BM (blue) or PB (red) sorted by frequency of mutation. **(B)** Correlation between BM VAF and PB VAF of individual mutations. **(C)** Bland-Altman plot showing the comparison between BM VAF and PB VAF of individual mutations. VAF: variant allele frequency.


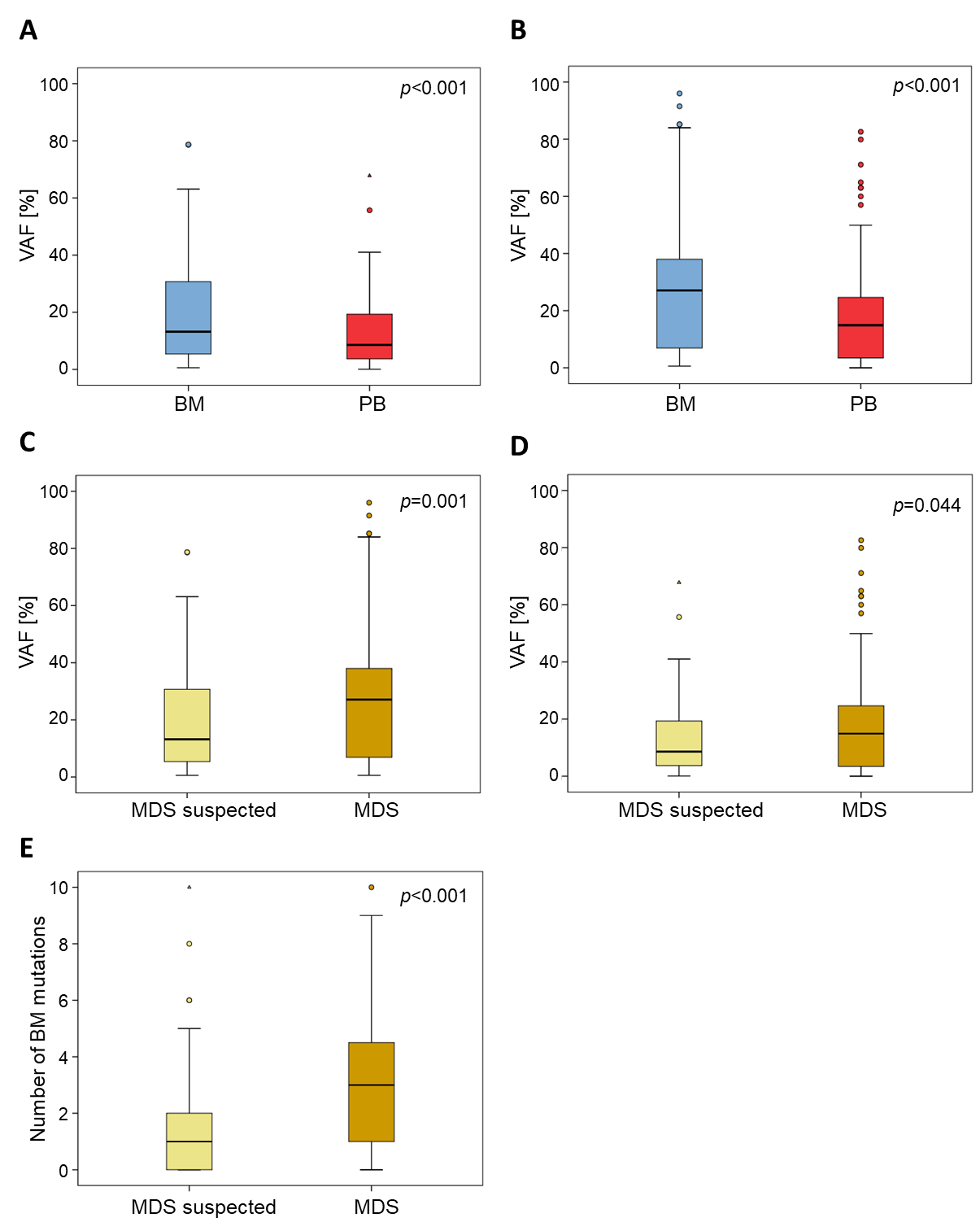


**Supplementary Figure S4: Comparison of variant allele frequency of detected mutations.** Distribution and median VAF of mutations detected in BM (blue) or PB (red) in patients with suspected MDS **(A)** and patients with confirmed MDS **(B)**. Distribution and median VAF of mutations detected in BM **(C)** and PB **(D)** comparing patients with suspected MDS (yellow) with patients having a confirmed MDS diagnosis (brown). **(E)** Distribution and number of BM mutations comparing patients with suspected (yellow) and confirmed (brown) MDS diagnosis. VAF: variant allele frequency.


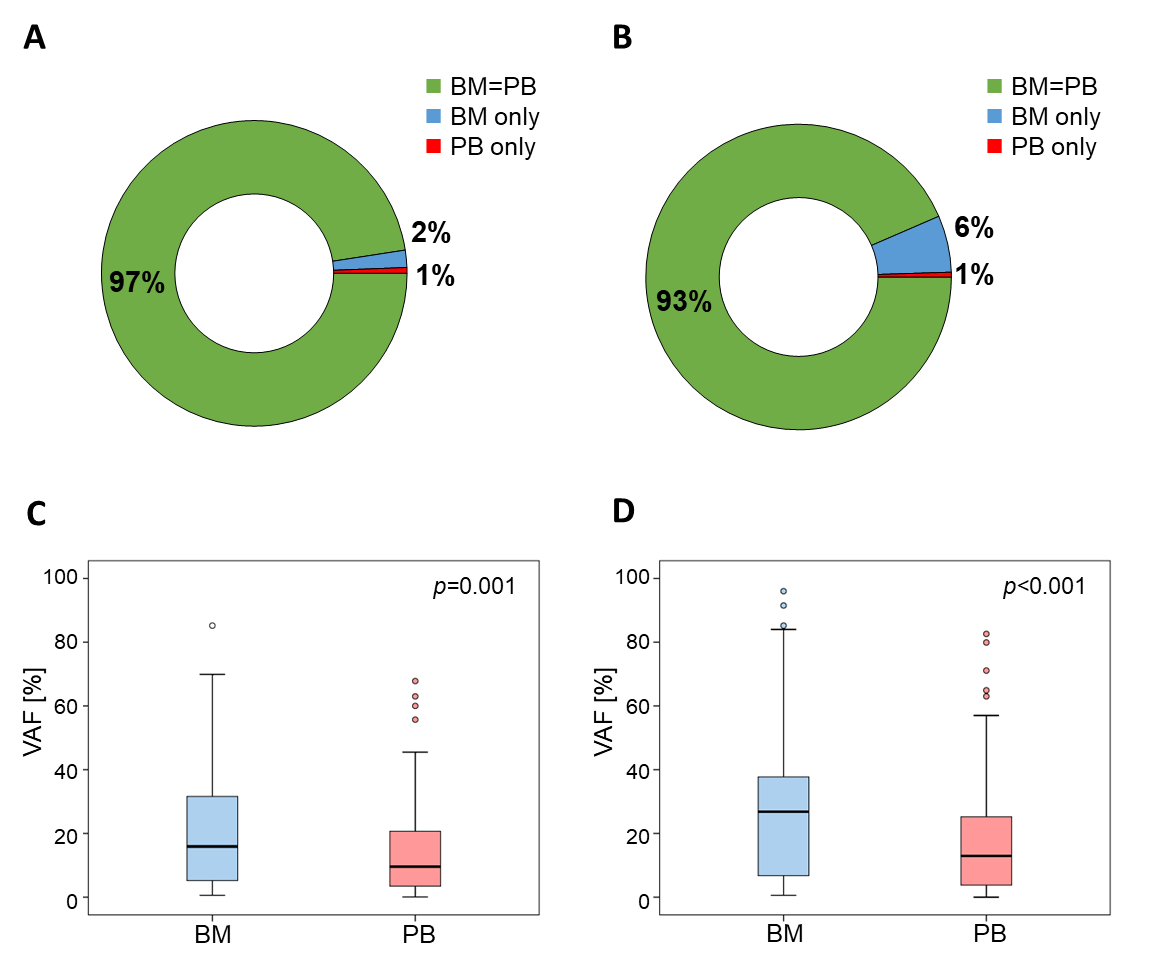


**Supplementary Figure S5: Individual mutation detection with respect to sampling time interval.** Concordance of mutation detection between BM and PB for patients with BM and PB sampled on the same day (**(A)** 164 mutations in 103 patients) or within 2 weeks (**(B)** 200 mutations in 97 patients). Green: concordant results (BM=PB). Distribution and median VAF of mutations detected in BM (blue) or PB (red) in patients with BM and PB sampled on the same day **(C)** or within 2 weeks **(D)**.

**
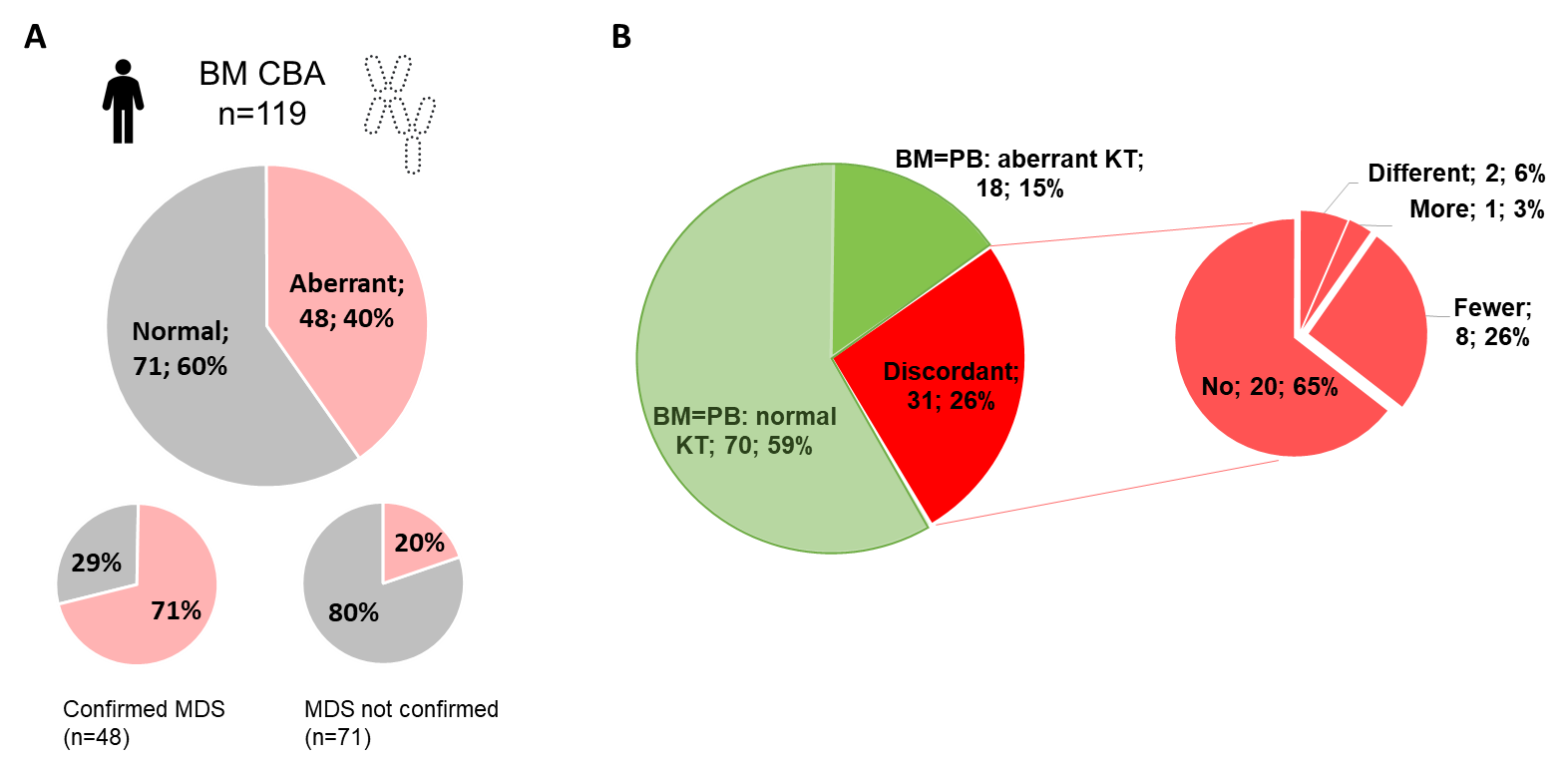
**

**Supplementary Figure S6: Cytogenetic analysis of BM and PB in 119 patients. (A)** Distribution of patients with normal or aberrant karyotype based on BM chromosome banding analysis (CBA) for all 119 patients (upper chart) and grouped into patients with and without confirmed MDS diagnosis separately (lower charts). **(B)**  Comparison of cytogenetic clonality detection in BM and PB of the 119 patients showing concordant (green) and discordant (red) results. KT: karyotype. Discordant patients are further sub-characterized into whether PB showed no, fewer, more or different cytogenetic abnormalities.

**
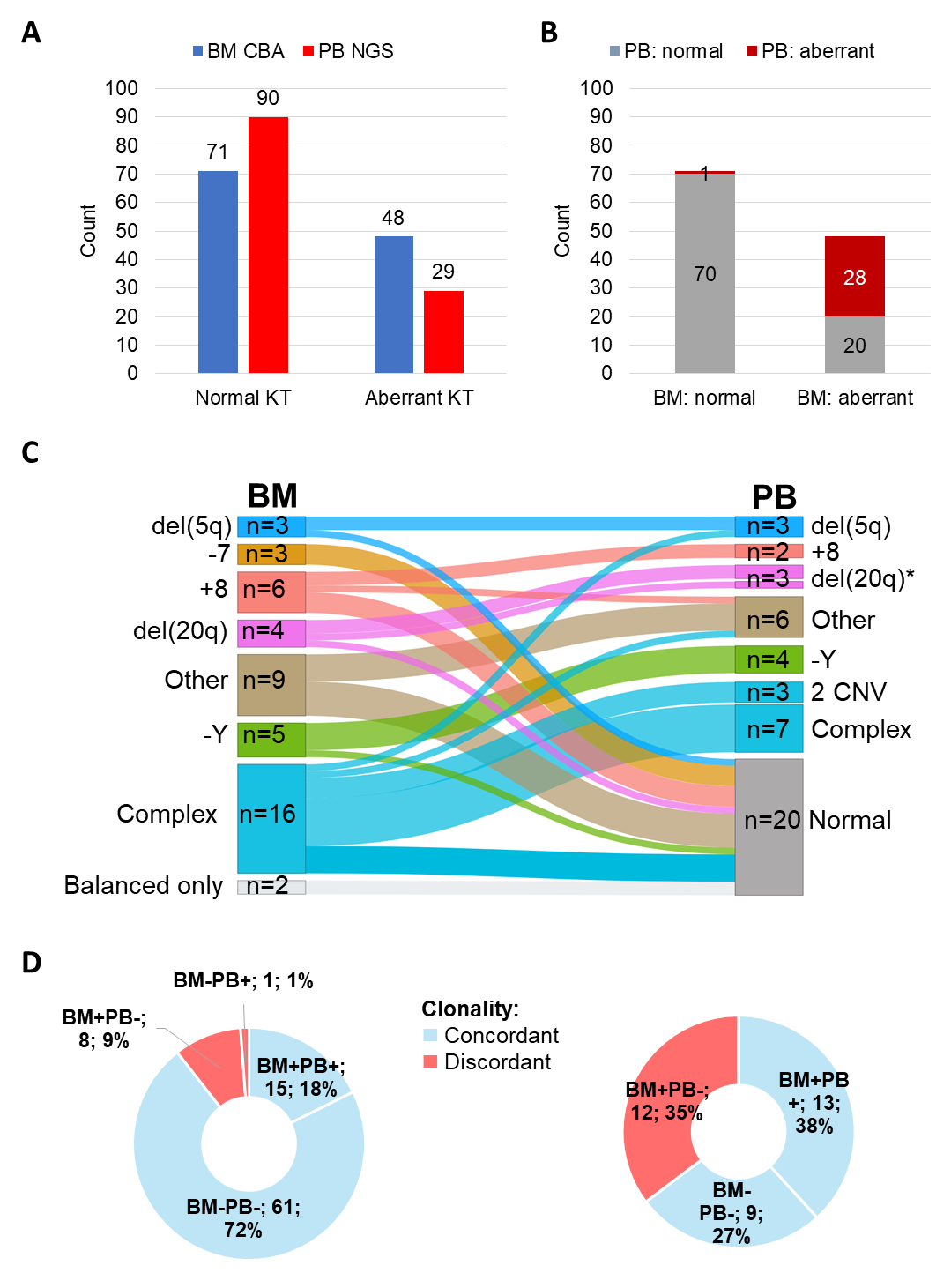
**

**Supplementary Figure S7: Detailed comparison of cytogenetic analysis between BM and PB. (A)** Karyotype (KT) analysis (normal vs. aberrant) for all 119 patients comparing BM chromosome banding analysis (CBA; blue) and PB NGS (red). **(B)** KT analysis for all 119 patients comparing BM CBA result with PB NGS result (PB normal KT: grey; PB aberrant KT: dark red). **(C)** Relationship between BM (left) and PB (right) comparing type of chromosomal abnormalities for cases with aberrant BM KT (n=48). CNV: copy number variation; *1/3 with additional PB CNV. **(D)** Comparison of cytogenetic clonality detection in BM and PB for patients with BM and PB sampled on the same day (n=85; left) or within 2 weeks (n=34; right). “+”: positive; ”–“: negative.

**
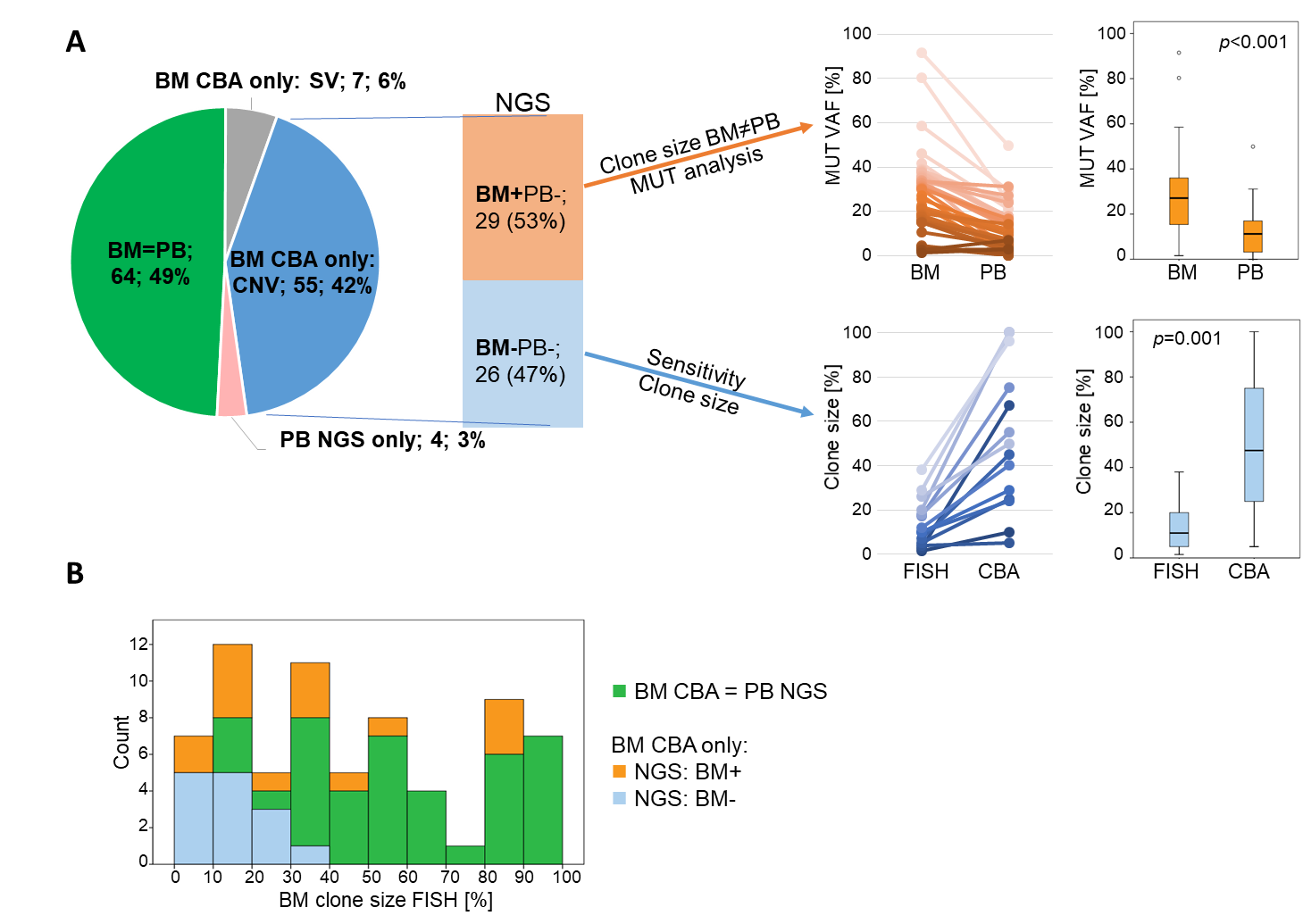
**

**Supplementary Figure S8: Detailed analysis of all detected chromosomal abnormalities. (A)** Pie chart represents concordance of chromosomal abnormality detection (n=130) comparing BM chromosome banding analysis (CBA) and PB NGS of 119 patients. BM=PB: concordant; SV: structural variation. Bar chart (middle) shows whether copy number variations (CNV) detected by BM CBA only (n=55; blue part of pie chart) were detected in BM with NGS; “+”: positive/orange; ”–“: negative/light blue. Clone sizes of NGS BM+ cases (orange) significantly differ between BM and PB analyzing the variant allele frequencies (VAF) of mutations (MUT) in BM and PB of these cases (orange graphs). Clone sizes of NGS BM- cases (light blue) significantly differ in BM between CBA and FISH (light blue graphs). **(B)** Histogram showing the BM clone size measured by percentage of aberrant FISH interphase nuclei of chromosomal abnormalities and their detection in BM and PB. Concordant chromosomal abnormalities detected in BM by CBA and PB by NGS are shown in green. Discordant chromosomal abnormalities (BM CBA only) were categorized whether they were also detected by NGS in BM (orange) or not (light blue). “+”: positive; ”–“: negative.


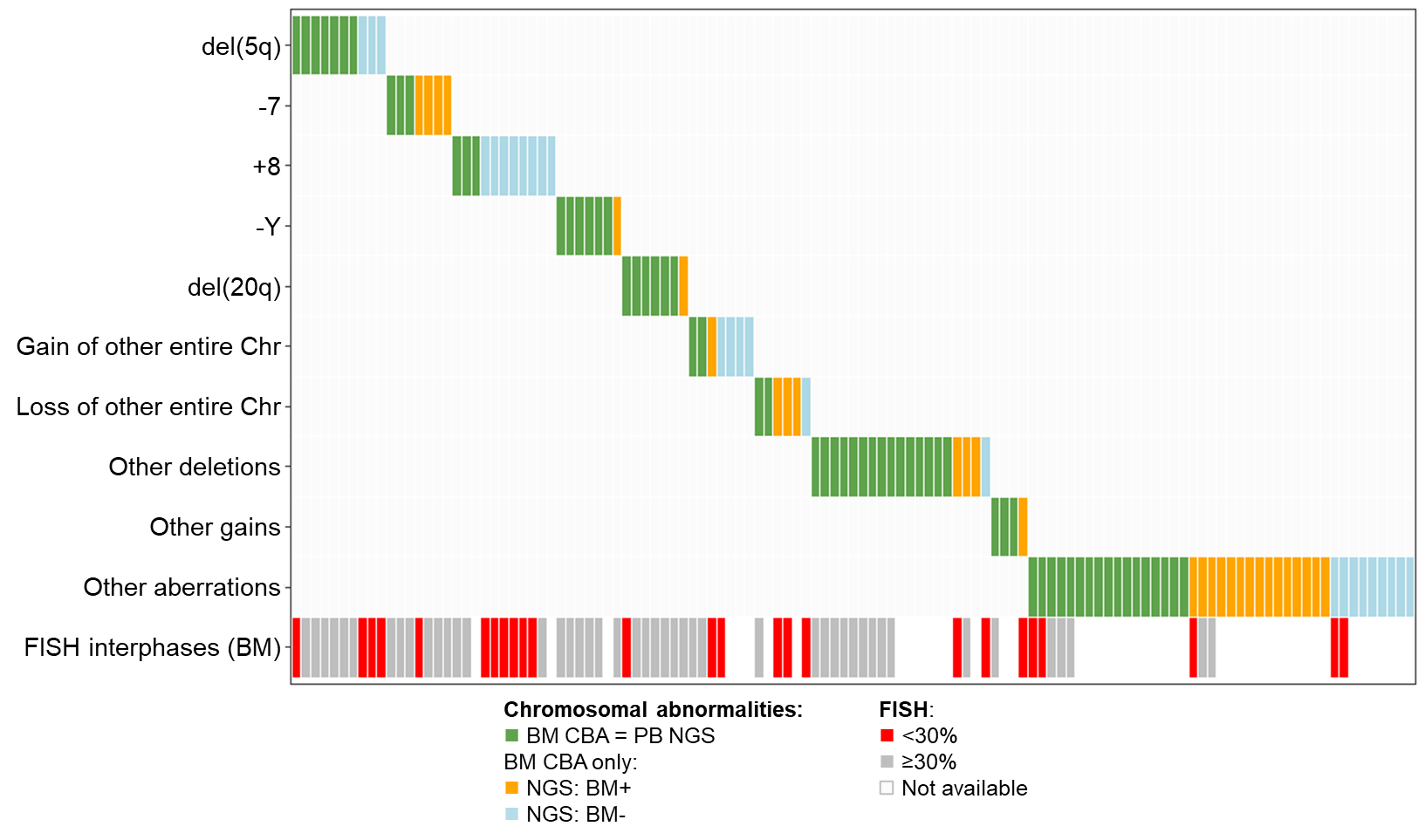


**Supplementary Figure S9: Analysis of specific chromosomal abnormalities comparing BM and PB.** Illustration of all chromosomal abnormalities (n=119, not including PB NGS only aberrations and structural variations detected in BM), each column represents one abnormality. Chromosomal abnormalities were either concordant (green) or discordant (orange and light blue) between BM CBA and PB NGS. Discordant chromosomal abnormalities (BM CBA only) were categorized whether they were also detected by NGS in BM (orange) or not (light blue). “+”: positive; ”–“: negative. If available, for each abnormality the percentage (<30% or ≥30%) of aberrant interphase nuclei detected by FISH in BM is shown in the last row. Chr: chromosome.


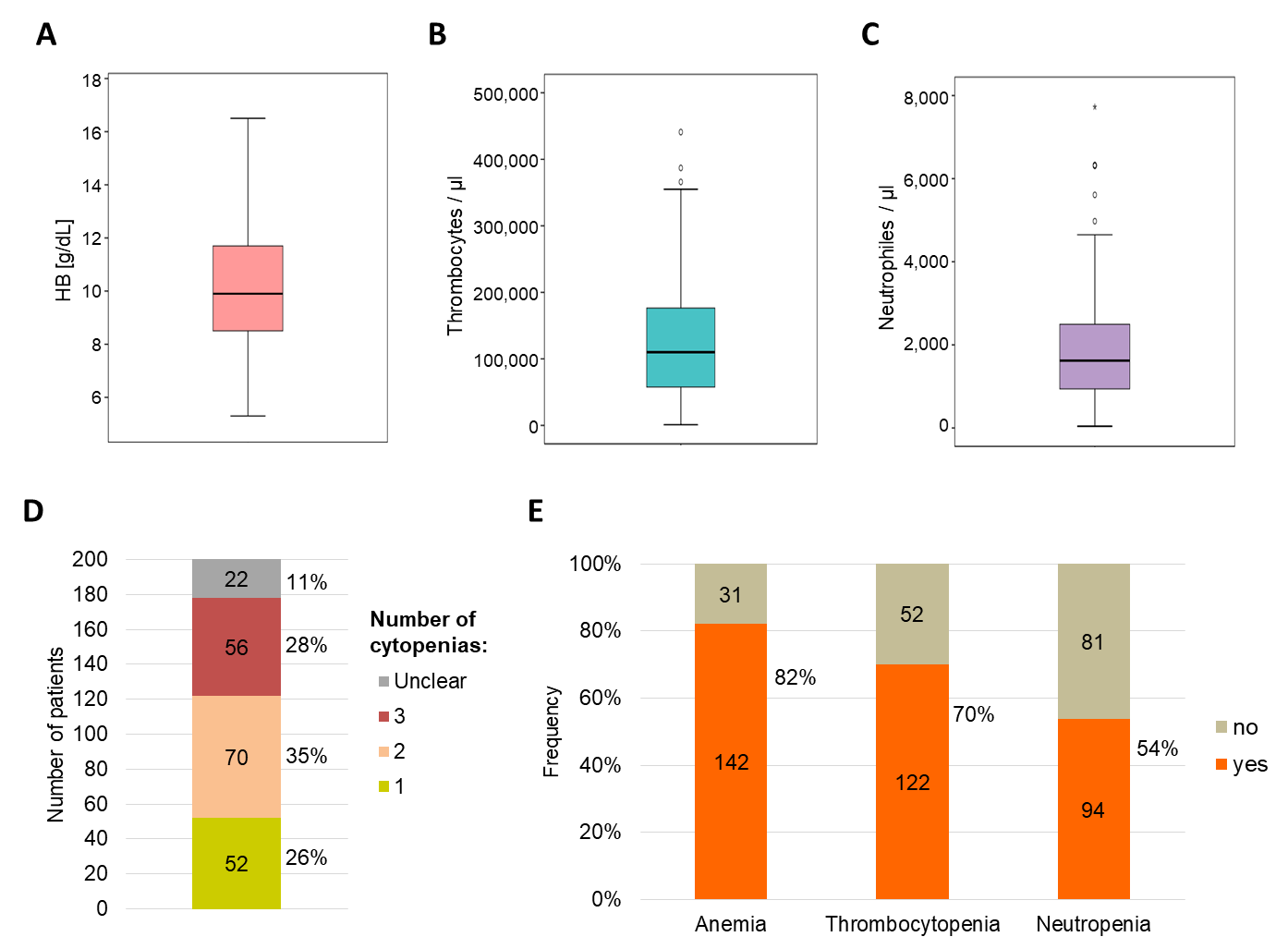


**Supplementary Figure S10: Blood parameter analysis** **of all 200 patients. (A)** Boxplot showing the distribution of hemoglobin (HB) values. **(B)** Boxplot showing the distribution of thrombocytes. **(C)** Boxplot showing the distribution of neutrophiles. **(D)** Number of cytopenias per patient. **(E)** Type of cytopenia across all patients.

**
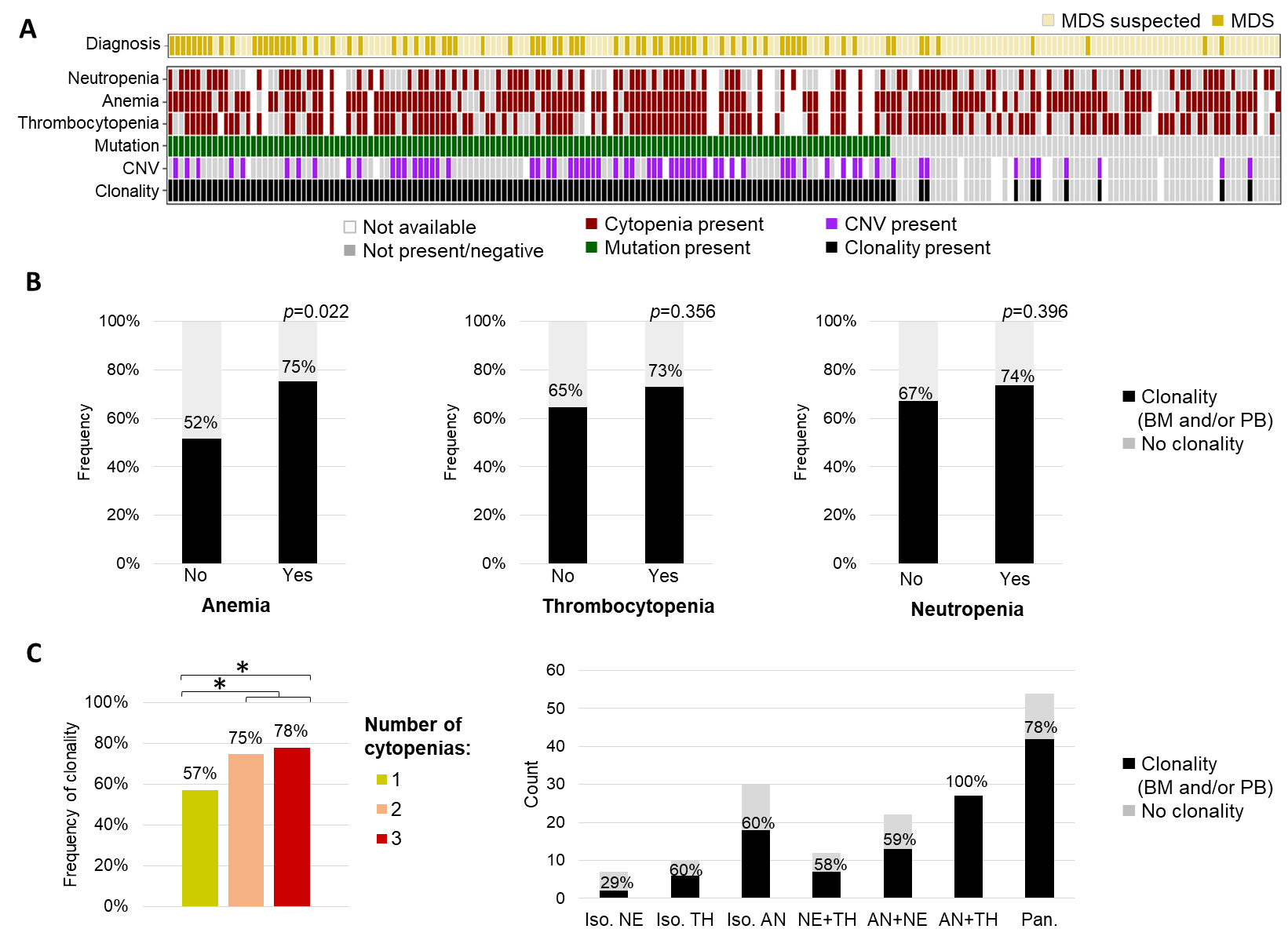
**

**Supplementary Figure S11: Association between cytopenia and clonality. (A)** Illustration of type of cytopenia and type of clonality (mutation or copy number variation/CNV detected in BM and/or PB), each column represents one patient (n=200). **(B)** Frequency of clonality dependent on presence of anemia (left), thrombocytopenia (middle) or neutropenia (right). **(C)** Frequency of clonality dependent on number of cytopenias in general (left; * *p*<0.05) and on specific combinations of cytopenias (right). Iso.: isolated; NE: neutropenia; TH: thrombocytopenia; AN: anemia; Pan.: pancytopenia.

**
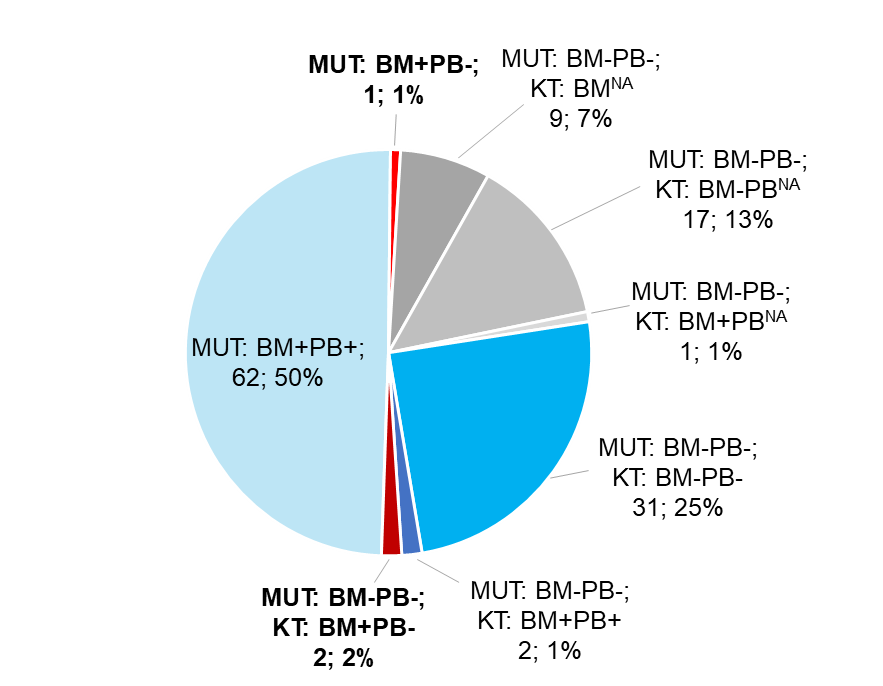
**

**Supplementary Figure S12: Clonality detection in patients without confirmed MDS diagnosis.** Evaluation of detection of clonality comparing BM and PB of patients with suspected MDS (n=125). MUT: mutation; “+”: positive/aberrant; ”–“: negative/normal; KT: karyotype; NA: not available; blue colors: concordant; red: discordant.


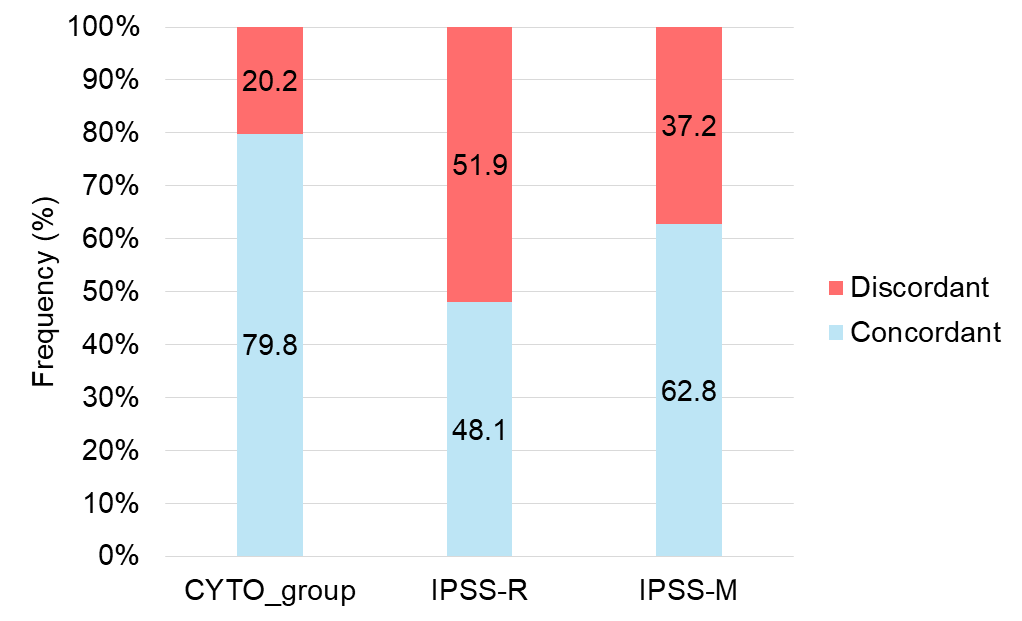


**Supplementary Figure S13: BM/PB concordance for the different risk models.** Evaluation of concordance between BM and PB regarding risk group assignment for the different models: IPSS-R cytogenetic risk group (CYTO_group), IPSS-R risk category and IPSS-M risk category.

**
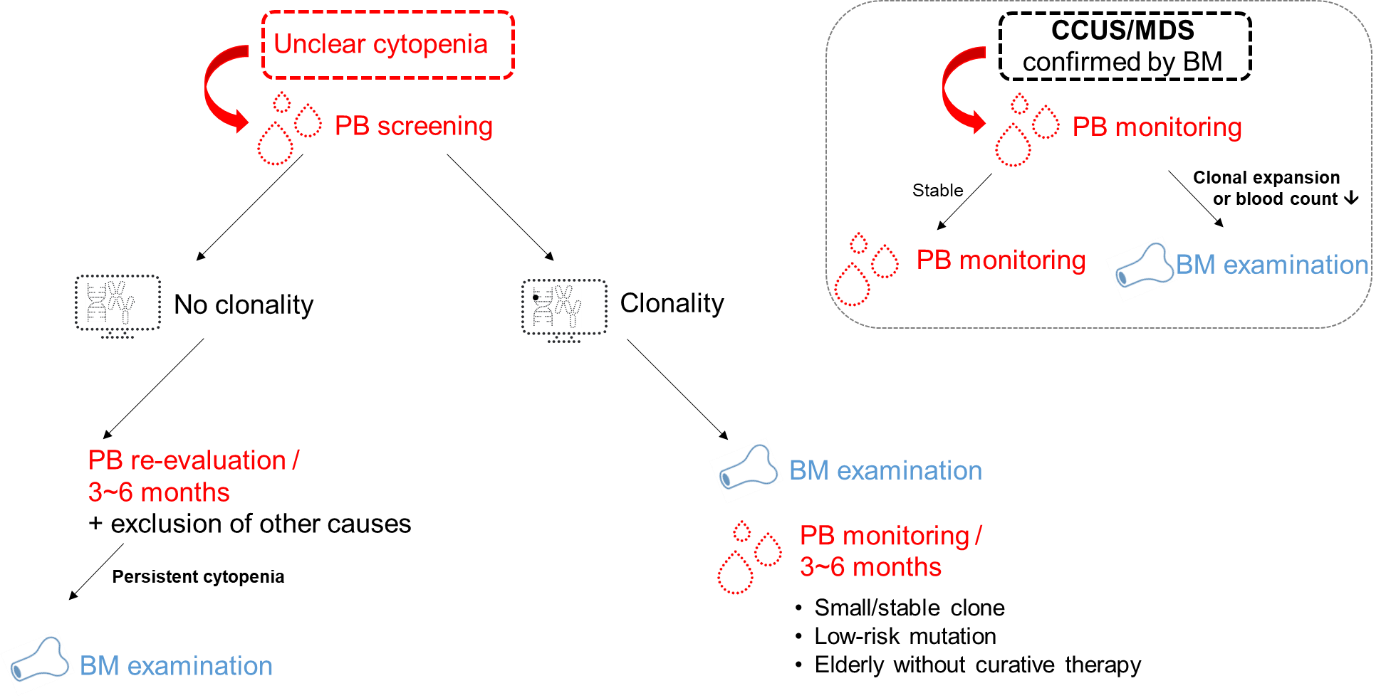
**

**Supplementary Figure S14: Suggested procedure using PB or BM in cytopenic patients.** At first PB should be screened in patients with unclear cytopenia (left). Depending on the absence or presence of clonality detection either PB or BM should be (re-)evaluated. In CCUS/MDS patients (upper right) PB might be used for monitoring the disease considering a BM evaluation only if the disease is getting worse (clonal expansion or worsening of blood counts).

1. Schoch C, Schnittger S, Bursch S, Gerstner D, Hochhaus A, Berger U, et al. Comparison of chromosome banding analysis, interphase- and hypermetaphase-FISH, qualitative and quantitative PCR for diagnosis and for follow-up in chronic myeloid leukemia: a study on 350 cases. Leukemia. 2002;16(1):53-9.

2. Haferlach T, Kern W, Schoch C, Hiddemann W, Sauerland MC. Morphologic dysplasia in acute myeloid leukemia: importance of granulocytic dysplasia. J Clin Oncol. 2003;21(15):3004-5.

3. Kern W, Voskova D, Schoch C, Hiddemann W, Schnittger S, Haferlach T. Determination of relapse risk based on assessment of minimal residual disease during complete remission by multiparameter flow cytometry in unselected patients with acute myeloid leukemia. Blood. 2004;104(10):3078-85.

4. Maierhofer A, Mehta N, Chisholm RA, Hutter S, Baer C, Nadarajah N, et al. The clinical and genomic landscape of patients with DDX41 variants identified during diagnostic sequencing. Blood Adv. 2023; doi:10.1182/bloodadvances.2023011389.

5. Robinson JT, Thorvaldsdóttir H, Winckler W, Guttman M, Lander ES, Getz G, et al. Integrative genomics viewer. Nat Biotechnol. 2011;29(1):24-6.

6. Li MM, Datto M, Duncavage EJ, Kulkarni S, Lindeman NI, Roy S, et al. Standards and Guidelines for the Interpretation and Reporting of Sequence Variants in Cancer: A Joint Consensus Recommendation of the Association for Molecular Pathology, American Society of Clinical Oncology, and College of American Pathologists. J Mol Diagn. 2017;19(1):4-23.

7. Talevich E, Shain AH, Botton T, Bastian BC. CNVkit: Genome-Wide Copy Number Detection and Visualization from Targeted DNA Sequencing. PLoS Comput Biol. 2016;12(4):e1004873.

8. Greenberg PL, Tuechler H, Schanz J, Sanz G, Garcia-Manero G, Sole F, et al. Revised International Prognostic Scoring System (IPSS-R) for myelodysplastic syndromes. Blood. 2012;120(12):2454-65.

9. Bernard E, Tuechler H, Greenberg Peter L, Hasserjian Robert P, Arango Ossa Juan E, Nannya Y, et al. Molecular International Prognostic Scoring System for Myelodysplastic Syndromes. NEJM Evidence. 2022;1(7):EVIDoa2200008.
